# Evaluation of *Mycobacterium tuberculosis* lipoarabinomannan antigen assay and rapid serology blood test for the diagnosis of bovine tuberculosis in Ethiopia

**DOI:** 10.1186/s12917-019-2114-3

**Published:** 2019-10-22

**Authors:** Aboma Zewude, Temesgen Mohammed, Lemma Terfassa, W. Garrett Hunt, Xueliang Pan, Joan Miquel Balada-Llasat, Wondwossen Gebreyes, Jordi B. Torrelles, Shu-Hua Wang, Gobena Ameni

**Affiliations:** 1 Aklilu Lemma Institute of Pathobiology, Addis Ababa University, Addis Ababa, Ethiopia; 2 Nationwide Children’s Hospital, Section of Infectious Diseases, College of Medicine, The Ohio State University, Columbus, OH USA; 3 Center for Biostatistics, College of Medicine, The Ohio State University, Columbus, OH USA; 4 Department of Pathology, College of Medicine, The Ohio State University, Columbus, OH USA; 5 Global One Health initiative (GOHi), The Ohio State University, Columbus, OH USA; 6 Veterinary Preventive Medicine, College of Veterinary Medicine, The Ohio State University, Columbus, OH USA; 7 Department of Microbial Infection and Immunity, College of Medicine, The Ohio State University, Columbus, OH USA; 8 Present Address: Texas Biomedical Research Institute, San Antonio, TX USA; 9 Division of Infectious Diseases, Department of Internal Medicine, College of Medicine, The Ohio State University, Columbus, OH USA

**Keywords:** TB LAM antigen, LIONEX animal TB rapid, Bovine tuberculosis, Diagnostic performance, Ethiopia

## Abstract

**Background:**

Bovine tuberculosis (bTB) is prevalent in dairy cattle in Ethiopia. Currently used diagnostic tools such as the single intradermal comparative tuberculin test (SICTT) are time consuming and labor intensive. A rapid, easy-to-use and cost-effective diagnostic test would greatly contribute to the control of bTB in developing countries like Ethiopia. In the present study, two point-of-care diagnostic tests were evaluated for the detection of bTB: LIONEX® Animal TB Rapid test, a membrane-based test for the detection of antibodies to *Mycobacterium bovis* in blood and ALERE® Determine TB Lipoarabinomannan (LAM) Ag, an immunoassay for the detection of lipoarabinomannan (LAM) antigen (Ag) of mycobacteria in urine. A combination of the SICTT and gamma interferon (IFN-γ) test was used as the gold standard for the validation of these point-of-care tests, as it was not feasible to slaughter the study animals to carry out the historical gold standard of mycobacterial culture. A total of 175 heads of cattle having three different bTB infection categories (positive SICTT, negative SICTT, and unknown SICTT status) were used for this study.

**Result:**

The sensitivity and specificity of TB LAM Ag were 72.2% (95% CI = 62.2, 80.4) and 98.8% (95% CI = 93.6, 99.7), respectively, while the sensitivity and specificity of the LIONEX Animal TB rapid test assay were 54% (95% CI = 44.1 64.3) and 98.8% (95% CI = 93.6, 99.7) respectively. The agreement between TB LAM Ag and SICTT was higher (κ = 0.85; 95% CI = 0.65–0.94) than between TB LAM Ag and IFN-γ (κ = 0.67; 95% CI = 0.52–0.81). The agreement between LIONEX Animals TB Rapid blood test and SICTT was substantial, (κ = 0.63; 95% CI = 0.49–0.77) while the agreement between LIONEX Animal TB rapid blood test and IFN-γ test was moderate (κ = 0.53; 95% CI = 0.40–0.67). Analysis of receiver operating curve (ROC) indicated that the area under the ROC curve (AUC) for TB LAM Ag was 0.85 (95% CI = 0.79–0.91) while it was 0.76 (95% CI; =0.69–0.83) for LIONEX Animal TB rapid test assay.

**Conclusion:**

This study showed that TB LAM Ag had a better diagnostic performance and could potentially be used as ancillary either to SICTT or IFN-γ test for diagnosis of bTB.

## Background

Bovine tuberculosis (bTB) is a chronic progressive disease of mammals caused primarily by *Mycobacterium bovis*, a member of the *M. tuberculosis* complex (TBC), and is characterized by the development of granulomatous lesions (tubercles) in the lymph nodes, lungs and other tissues. *M. bovis* can be transmitted from animal to human and is estimated to cause approximately 10–15% of human TB cases in low- and middle-income countries (LMIC) [1]. The economic impact of bovine TB is significant and accounts for over $3 billion in annual expenditures worldwide [2]. Although high-income countries have been implementing the test-and-slaughter control policy, LMIC are unable to support the cost of a test-and-slaughter policy. Consequently, in Africa, where 85% of cattle and 82% of the human population reside, there is absent or only a partial bTB control policy [3].

In Ethiopia, studies have shown that there is a widespread but variable occurrence of bTB throughout the country based on cattle breed and dairy farm conditions [4, 5]. A review and meta-analysis of the prevalence of bTB over 16 years (2000–2016) showed a pooled prevalence of 5.8% [5]. Higher prevalence (21.6%) was observed in the Holstein-Friesian breed compared to a low prevalence (4.1%) recorded in the zebu breed. Furthermore, the same review showed higher prevalence (16.6%) in intensive farms as compared to low prevalence (4.6%) in extensive farms.

Because of its chronic nature, bTB is difficult to detect clinically until its late stage [6]. Furthermore, the presently used diagnostic tests have constraints that compromise their diagnostic performance. The two most widely used methods are 1) the single intradermal comparative tuberculin test (SICTT), based on cutaneous measurement of a delayed-type hypersensitivity response and 2) the interferon-gamma (IFN-γ) release assay, an enzyme-linked immunosorbent assay that measures the production of IFN-γ from activated whole blood incubated with *M. bovis*-specific antigens. Attempts to evaluate bTB diagnostic tests in naturally infected cattle are rare and complicated by the absence of the status of the truly diseased animals [7–9]. The constraints associated with SICTT include poor specificity, repeat visits needed for skin test placement and reading, desensitization after tuberculin inoculation, false negativity in late pregnancy and early parturition, and cross-reactivity with other nontuberculous mycobacteria antigens [10]. The IFN-γ assay also has constraints that limit its diagnostic potential, including low specificity particularly in young cattle due to underdeveloped immune system [10], the need to incubate unfrozen blood samples within 18 h of collection, and high cost of the test for LMIC [10]. Hence, it is paramount to search for diagnostic tests that are rapid, cost-effective and can easy to use in developing countries such as Ethiopia.

Currently, two point-of-care diagnostic tests are available for detecting TBC, the ALERE® Determine LAM urine test and the LIONEX® Animal TB Rapid blood test [11–13]. Mannose capped liporabinomannan (LAM) is the major surface antigen of the *M. bovis* cell wall and may account for up to 15% of the total bacterial weight. In active TBC disease, LAM is released from both metabolically active and degrading bacteria. LAM is subsequently cleared through the kidneys and can be detected in urine. Detection of LAM in the urine can be used for the diagnosis of bTB using the LAM kit that is currently used for the diagnosis of human TBC disease in individuals with HIV/AIDS (ALERE® Determine LAM, United States) [11, 12]. LIONEX® Animal TB Rapid test has developed a number of highly purified mycobacterial antigens, which can be used for sero-diagnosis of TB in whole blood, serum, or plasma samples from cattle or other mammals (LIONEX® Animal TB Rapid test, Germany) [13].

In this study the LIONEX Animal-TB Rapid test and the ALERE Determine TB LAM Ag test were evaluated for sensitivity, specificity and agreement (Kappa statistic) in comparison to SICTT and IFN-γ. Cattle obtained from herds with known bTB infection were enrolled in the study and used for the validation of the two tests.

## Result

### Antibody detection test

In the present study, a total of 175 animals from the three different herd categories were enrolled: Group one consisted of 51 cattle with known positive SICTT and IFN-γ; Group two consisted of 64 cattle with negative SICTT and/ or IFN-γ; and Group three with 60 cattle with unknown SICTT status. Each animal underwent testing by SICTT, IFN-γ, TB LAM Ag test and LIONEX Animal TB rapid test. On the basis of SICTT, 45% (79/175) of the animals were positive for bTB. These 79 animals consisted of all 51 animals from a known TB infected herd (group one), 3 from the previously known TB negative herd (group two) and 25 animals from the unknown bTB status herd (group three) (Table 1).

**Table 1:** Agreement of TB LAM Ag or LIONEX animal TB tests with SICTT in detecting bovine TB

Type of test	SICTT			Kappa statistics 95% Confidence Interval
	Positive	Negative	Total	
LAM TB Ag				
Positive	64	2	66	0.85 (0.65–0.94)
Negative	15	94	109	
Total	79	96	175	Strong agreement
LIONEX Animal TB				
Positive	49	1	50	0.63(0.49–0.76)
Negative	30	95	125	
Total	79	96	175	Moderate Agreement

*TB* Tuberculosis, *SICTT* Single intradermal comparative tuberculin test, *LAM* Lipoarabinomannan, *IFN-γ* Gamma interferon, *Ag* Antigen

### Agreement of TB LAM Ag or LIONEX animal TB rapid test with the SICTT

The results of both the **ALERE Determine** TB LAM Antigen (Ag) urine test and LIONEX Animal TB rapid blood test were read visually. From the total of 79 SICTT positive animals, 66 animals were positive by the TB LAM Antigen test, comprised of 37 from TBC-infected cattle, 26 from herds with unknown TB status and 3 from the “infection-free” herd. Fifty cattle were found to be positive by the LIONEX Animal TB rapid blood test: 28 animals from the herd known to be TBC-infected herd and 22 from herds with unknown TB status. Forty-nine of the 50 animals with positive LIONEX Animal TB rapid blood test animals also tested positive by SICTT (Table 1). The discordant test result between positive LIONEX Animal TB rapid blood test and negative SICTT was from an animal with unknown TB status. All of the 96 SICTT negative cattle had negative TB LAM Antigen as well as negative LIONEX Animal TB rapid blood test. As seen in Table 1, the test agreement between SICTT and TB LAM Ag test was strong (κ = 0.85 95% CI = 0.65–0.94). There was also good agreement between the LIONEX animal TB rapid test and SICTT (κ = 0.63; 95%CI = 0.49–0.76).

### Agreement of TB LAM Ag or LIONEX animal TB rapid test with the IFN-γ test

A total of 86 animals that were negative for the IFN-γ test were also negative by the LIONEX Animal TB rapid blood test and the TB LAM Antigen test. However, of the 64 cattle from the known negative herd (group two), one animal was positive by the LIONEX Animal TB rapid blood test and the TB LAM Antigen tests. From 89 IFN-γ assay positive animals, 63 were positive by the TB LAM antigen: 36/63 from the herd with known TB infection (group one), 3/63 from the herd known to be negative (group two), and 26/63 from the herd with unknown status (group three) (Table 2). The agreement between TB LAM Ag and IFN-γ test was substantial (κ = 0.67; 95% CI = 0.52–0.81), while the agreement between LIONEX Animal Ag test and IFN-γ test was moderate (κ = 0.53; 95%CI = 0.40–0.66).

**Table 2:** Agreement of tuberculosis (TB) LAM Ag or LIONEX animal TB tests with IFN-γ test in detecting bovine TB

Type of test	IFN-γ test PPD (B-A)			Kappa statistics 95% Confidence Interval
	Positive	Negative	Total	
TB LAM Ag				
Positive	63	3	66	0.67 (0.52–0.81)
Negative	26	83	109	
Total	89	86	175	Substantial agreement
LIONEX Animal TB				
Positive	49	1	50	0.53 (0.40–0.66)
Negative	40	85	125	
Total	89	86	175	Moderate agreement

*TB* Tuberculosis, *LAM* Lipoarabinomannan, *IFN-γ, SICTT* Single intradermal comparative tuberculin test

### Sensitivity and specificity of TB LAM Ag and LIONEX animal TB rapid tests

The sensitivity and specificity of the TB LAM Ag and LIONEX Animal TB rapid tests were estimated by considering the combination of SICTT and IFN-γ as a gold standard. Out of 175 tested animals, 90 were positive for both SICTT and the IFN-γ test. Of these 90 animals, 65 animals tested positive, 25 negative, and one falsely negative. Accordingly, the sensitivity of TB LAM Ag test was 72%, while its specificity was 98.8% (Table 3). The sensitivity and specificity of the LIONEX Animal TB rapid test were 54.4 and 98.8%, respectively (Table 3).

**Table 3:** Diagnostic performance (sensitivity and specificity) of TB LAM Ag or LIONEX animal TB rapid test

Type of test	Combined of SICTT and IFN-γ tests			Sensitivity Specificity
	Positive	Negative	Total	
LAM TB Ag				
Positive	65	1	66	72.2%(62.2,80.42) 98.8% (93.6, 99.79)
Negative	25	84	109	
Total	90	85	175	
LIONEX Animal TB				
Positive	49	1	50	54%(44.1, 64.3) 98.9%(93.6, 99.79)
Negative	41	84	125	
Total	90	85	175	

*TB* Tuberculosis, *LAM* Lipoarabinomannan, *IFN-γ, SICTT* Single intradermal comparative tuberculin test

**Fig. 1:**
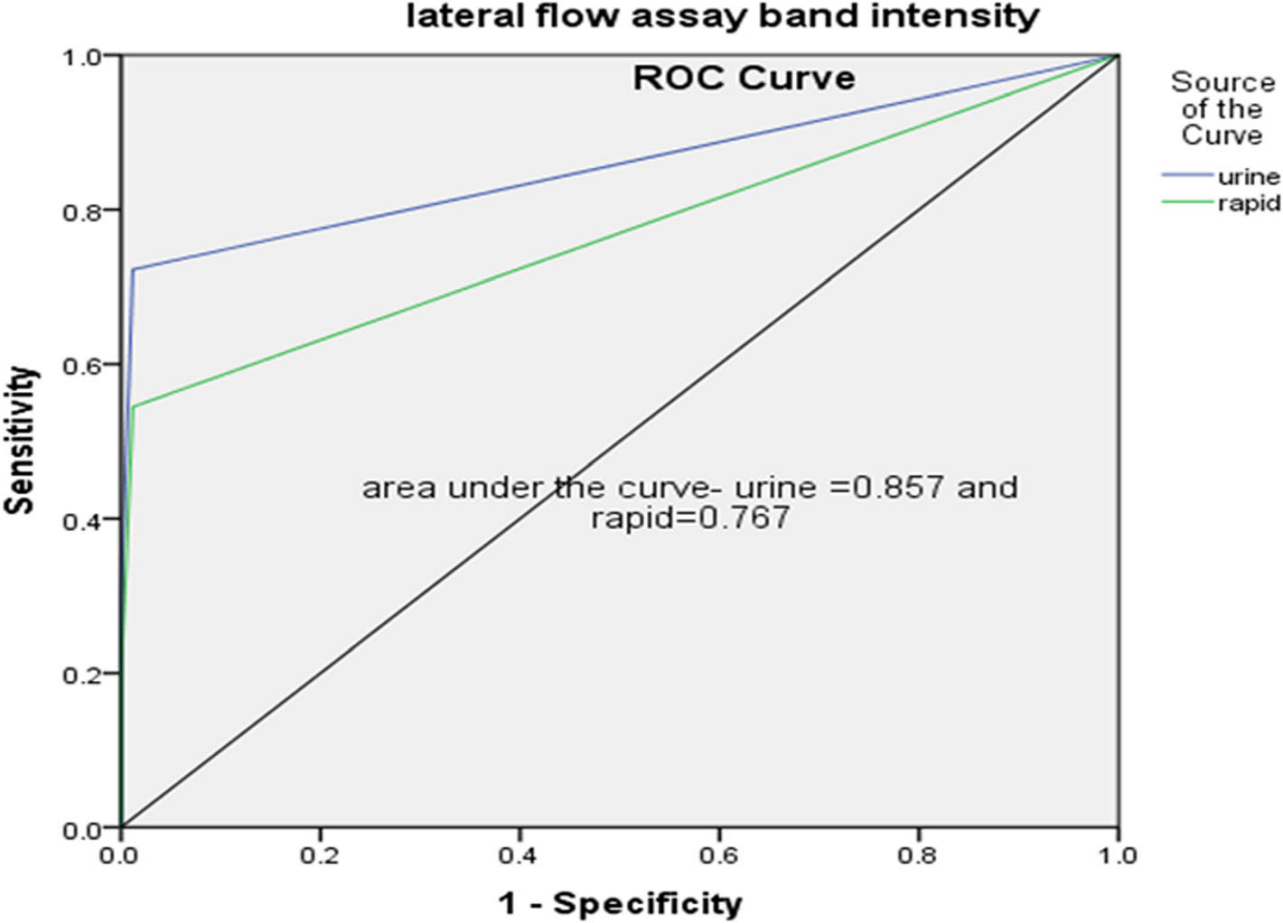
Receiver Operating Characteristics (ROC) curve for TB LAM Ag and LIONEX Animal TB rapid blood test. The area under ROC curve (AUC) of TB for TB LAM Ag test was 0.85 (95%CI = 0.79–0.91) and for the LIONEX Animal TB rapid blood test was 0.76 (95%CI; =0.69–0.83)

Receiver Operating Characteristics (ROC) analysis was performed for assessing the performances of the two tests. Accordingly, the area under ROC curve (AUC) of TB for TB LAM Ag test was 0.85 (95%CI = 0.79–0.91) while it was 0.76 (95%CI; =0.69–0.83) for the LIONEX Animal TB rapid blood test (Fig. 1).

## Discussion

In this preliminary study, diagnostic performance of the TB LAM Ag urine test and LIONEX Animal TB Rapid blood test was evaluated using the combined result of SICTT and IFN-γ test as a gold standard.

The test results of each assay were compared with those of the standard tests i.e. with those of the SICTT or with those of the IFN-γ test [14]. There was high concordance between the TB LAM Ag test and that of SICTT. Furthermore, the agreement between TB LAM Ag and IFN-γ test was substantial. The sensitivity and specificity of the TB LAM Ag were 72 and 98.8%, respectively. A similar finding was reported by Lamont et al. a study with a similar design that was conducted in California [15]. The result of a meta-analysis published by Minion et al. on human subjects showed that the sensitivity of the TB LAM Ag test ranged from 13 to 93% while its specificity ranged from 87 to 99%. Thus, the sensitivity and specificity of TB LAM Ag recorded by the present study were within the ranges reported by Minion et al. Our findings are encouraging and could suggest the potential use of TB LAM Ag test for the diagnosis of bTB. Technically, a test such as the TB LAM Ag that uses urine samples is preferable to one that relies on blood or serum. Bacterial metabolism and degraded cell wall shed into body fluids could be detected by TB LAM Ag, which implies presence of active TB disease [16].

Diagnostic performance of TB LAM Ag from urine of bTB-infected animals has been studied by different groups [15, 17]. In experimentally infected animals, evaluation of LAM excretion [17] indicated that LAM is more frequently detected in the first 7 to 14 days post-infection and then decreases onward. Cross-reactivity of the TB LAM Ag was also reported with no significant changes. The ability of the test to differentiate bTB infected and not infected animals was as good as observed in experimentally and naturally infected bovine serum [15].

Additionally, test agreement between the SICTT and LIONEX Animal TB rapid blood test was substantial, while the test agreement between IFN-γ test and LIONEX Animal TB rapid blood test was moderate. Furthermore, the sensitivity and specificity of LIONEX Animal TB rapid blood test were 54 and 98.8%, respectively. Thus, according to the results of this preliminary study, both the sensitivity and agreement of LIONEX Animal TB rapid blood test with either SICTT or IFN-γ test was inferior to those of the TB LAM Ag. LIONEX Animal TB rapid is new and its field evaluation is still ongoing in different geographic areas like Gonder, Ethiopia and with milk and urine specimens from Michigan, USA (unpublished data). LIONEX TB animal rapid kit failed to identify many SICTT-positive animals and had relatively lower sensitivity compared to the other serological tests [18, 19].

Because of a number of limitations of SICTT, including cross reactivity with environmental mycobacteria, animal desensitization after severe infection, long test performance time, and interference with vaccination [10], there remains a need to identify tests with better performance characteristics.

Serological assays could be additional tools to detect animals that do not react to cell mediated immune based tests [20]. Such methods could aim for detecting either mycobacterial specific antigens that are an immunologically active component or possibly mycobacterial specific antibodies. In general, serological tests perform better during the latter stages of infection [21, 22] as the immune response to bTB shifts from cellular to humoral [22]. It has been indicated that specificity of serological tests is higher and minimizes the proportion of false positive reactions in non-infected animals [23].

## Conclusion

In a low resource setting where infrastructure is limited, a rapid, field-deployable test which can improve diagnosis of bTB would allow for a decrease in zoonotic transmission and thereby improve public health. The TB LAM Ag had a better diagnostic performance than the LIONEX animal TB rapid blood test and could potentially be used as ancillary either to SICTT or IFN-γ test for the diagnosis of bTB. Additional studies in a real-time field setting to determine its utility and implementation capacity are needed. .

## Methods

### Study animals and settings

#### Animals

The study was conducted in central Ethiopia and 175 study animals were enrolled from different farms and divided into distinct groups: 1) group one, with 51 SICTTand IFN-γ positive animals recruited from the National Animal Health Diagnostic and Investigation Center (NAHDIC) in Sebeta; 2) group two, with 64 SICTT and/or IFN-γ-negative animals recruited from a farm located at Muke-Turi, in the North Shewa Zone of the Oromia Region; and 3) Group three: 60 animals with unknown status of bTB were recruited from Chancho and Sululta, North Shewa of the Oromia Region. Since there is no prevention and control strategy in Ethiopia for BTB at this time, the animals that tested positive were not removed from the farms. Farmers were provided with the results of tests as well as educational awareness in order to minimize the risk of transmission to other animals and human.

### Single intradermal comparative tuberculin test (SICTT)

Two sites on one side of the mid-neck 12 cm apart were shaved and the skin thickness was measured with a caliper. One site was injected intradermally with an aliquot of 0.1 ml 3000 IU/ml bovine purified protein derivative (PPD) (Prionics Leslystad BV, Lelystad Netherland). The second site was injected with 0.1 ml 3000Ul/ml avian PPD (Prionics Leslystad BV, Lelystad Netherland). After 72 h the skin thickness at injection site was measured. An animal was defined as a reactor if the bovine site reaction exceeded the avian site reaction by > 4 mm at 72 h [24].

#### LIONEX animal TB rapid blood test

The animal TB rapid test is a lateral flow immunochromatography and membrane-based screening test for the rapid detection of antibodies to *M. bovis* in sample plasma or serum from animals, developed by LIONEX Diagnostic and Therapeutic GmbH Company, Germany [13]. Whole blood was collected into a heparinized tube and transported to the Aklilu Lemma Institute of Pathobiology immunology lab. Plasma was harvested after centrifugation at 1500 rpm for 10 min, and then the samples were stored at -20^0^c until processed.

**Fig. 2:**
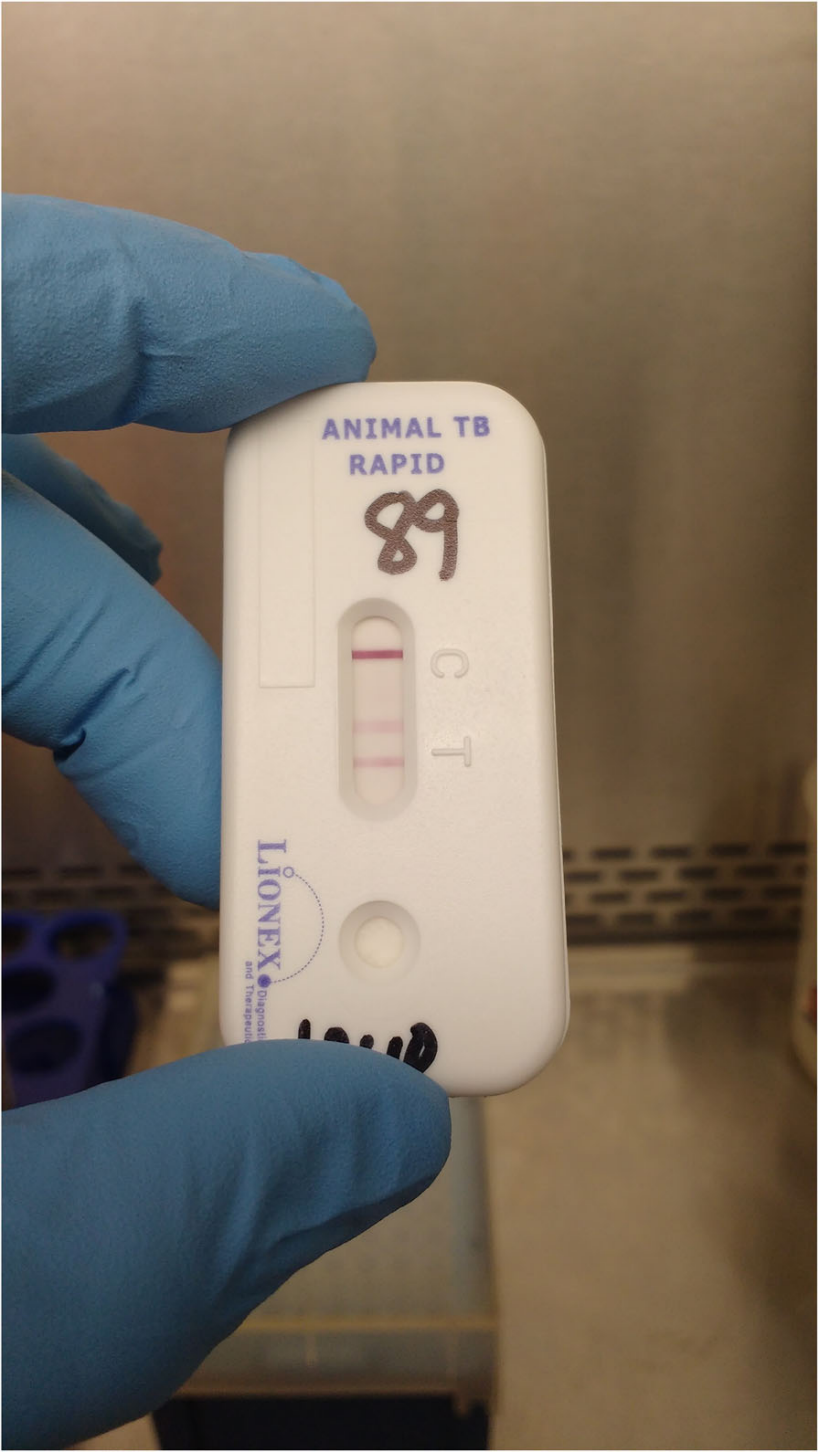
LIONEX Animal TB Rapid Blood Test. Positive LIONEX Animal TB rapid blood test is shown in the figure: One strong line appear in the control zone “C” and one or more test lines appear in the test zone “T”

On the test day the kit and the plasma samples were thawed, after which one drop (about 20 μl) of plasma was added to the designated test kit pad area. Two drops of the diluent buffer were added to the pad, mixed, and then kept at room temperature for 5 min. Next, 1 drop of diluent buffer was added to the solution and kept at room temperature for an additional 20 min, at which point the result was read. Interpretation of the result was based on manufacturer instructions, i.e., the test was considered positive when bands were observed at control bar C and sample bar T and negative if a band was observed only at control bar C. Intensity of the test band was graded per grading intensity scales supplied by the manufacturer: Zero for no band, 1 for faint band intensity, 2 for moderate intensity and 3 for strong test band intensity (Fig. 2).

#### ALERE determine LAM TB urine antigen test

**Fig. 3:**
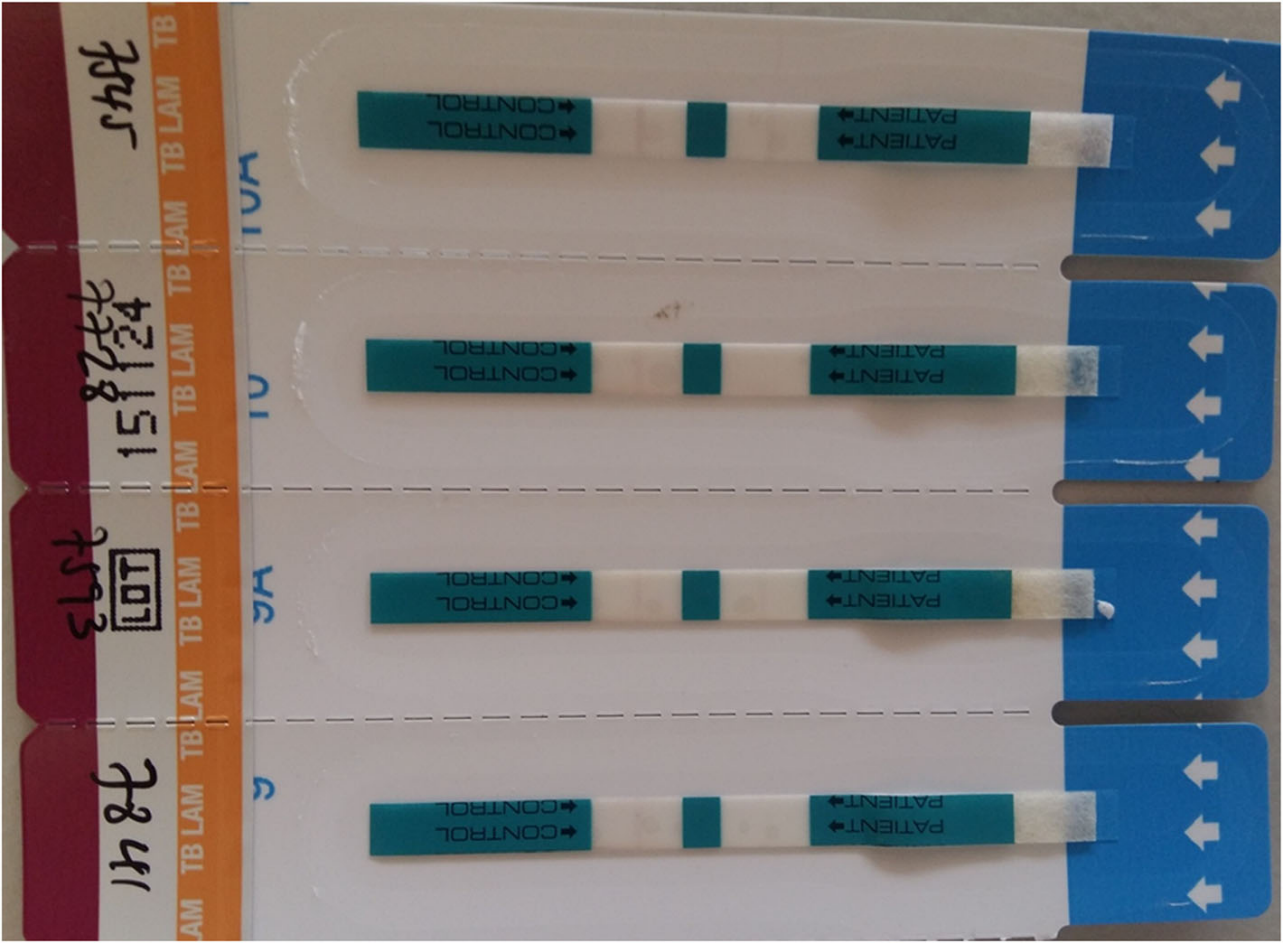
ALERE Determine LAM TB Urine Antigen Test. The TB LAM antigen urine test has Control Zone and Patient Zone. If a band is visualized in the Control zone and the patient zone, then the test is positive. If a band is visualized in the Control zone but no band is seen in the patient zone, then the test is negative

TB LAM Ag is an immunochromatographic test for the qualitative detection of LAM antigen of mycobacteria in urine, based on the use of highly purified antibodies specific to LAM (Fig. 3) [11]. The capture antibodies are adsorbed onto the nitrocellulose membrane of the test strip. This strip was originally designed for human TB detection, and we evaluated the assay for bTB infection diagnosis.

Urine samples were collected after manual stimulation of the vulva area and transported within 8 h of collection to the Aklilu Lemma Institute of Pathobiology (immunology laboratory). After removal of the protective foil cover from each TB LAM Ag test strip, 60 μl urine was added onto the sample pad on the test strip and kept at room temperature for at least 25 min to read the result. All test results were interpreted according to manufacturer instructions. Band intensity was graded per the manufacturer-supplied reference card: Zero for no band, 1 for faint band intensity, 2 for moderate band intensity, and 3 for strong test band intensity.

#### INF-γ test and whole blood culture

A 3 ml blood sample from each animal was collected into a heparinized tube after shaving and disinfecting the groove of the animal’s neck. All collected blood was transported to the Aklilu Lemma Institute of Pathobiology (ALIPB, immunology laboratory) within 8 h of collection. About 250 μl of whole blood was dispensed in duplicate into 96-well flat-bottom culture plates. Whole blood was stimulated using 25 μl of Avian-PPD and Bovine-PPD, giving a final assay concentration of 10 μg/ml in each well. Lectin from *Phytolacca americana* (pokeweed, Sigma) (5 μg/ml) was used as a positive control, and saline was used as a negative control, each dispensed at 25 μl into the corresponding wells. The culture was incubated at 37 °C in 5% CO_2_ atmosphere for 48 h and then the supernatant was harvested and frozen. IFN-γ in the supernatants were measured by an enzyme-linked immunosorbent assay using the Bovigam® test kit as per product insert (Prionics AG, Schlieren, Switzerland) [25]. The result was interpreted as per the manufacturer recommendation as positive, negative with the cut-off value of IFN- γ ≥0.1.

#### Data analysis

Test sensitivity (proportion of positive results found among infected animals), specificity (proportion of negative results among non- infected animals), positive predictive value (PPV) and accuracy (proportion of correct result among all tested animals) and their 95% confidence intervals (CI) were calculated using open Agreement between tests were evaluated using Kappa-statistic (k) using SPSS statistic version 24.

## Data Availability

The data used for analysis to support this study are mostly included in the published article. Additional data can be available from corresponding author on reasonable request.

## Abbreviations

Ag: Antigen
bTB: Bovine tuberculosis
HIV/AIDS: Human immunodeficiency virus; acquired immunodeficiency syndrome
IFN-ɤ: Gamma interferon
LAM: Lipoarabinomannan
LMIC: Low- and middle- income countries
PPD: Purified protein derivative
SICTT: Single intradermal comparative tuberculin test
TBC: *Mycobacterium tuberculosis* complex
